# YOLO-P: An efficient method for pear fast detection in complex orchard picking environment

**DOI:** 10.3389/fpls.2022.1089454

**Published:** 2023-01-04

**Authors:** Han Sun, Bingqing Wang, Jinlin Xue

**Affiliations:** ^1^College of Engineering, Nanjing Agricultural University, Nanjing, China; ^2^Agricultural Machinery Information Center, Department of Agriculture and Rural Affairs of Jiangsu Province, Nanjing, China

**Keywords:** deep learning, pear, fruit detection, YOLOv5, convolutional neural network

## Abstract

**Introduction:**

Fruit detection is one of the key functions of an automatic picking robot, but fruit detection accuracy is seriously decreased when fruits are against a disordered background and in the shade of other objects, as is commmon in a complex orchard environment.

**Methods:**

Here, an effective mode based on YOLOv5, namely YOLO-P, was proposed to detect pears quickly and accurately. Shuffle block was used to replace the Conv, Batch Norm, SiLU (CBS) structure of the second and third stages in the YOLOv5 backbone, while the inverted shuffle block was designed to replace the fourth stage’s CBS structure. The new backbone could extract features of pears from a long distance more efficiently. A convolutional block attention module (CBAM) was inserted into the reconstructed backbone to improve the robot’s ability to capture pears’ key features. Hard-Swish was used to replace the activation functions in other CBS structures in the whole YOLOv5 network. A weighted confidence loss function was designed to enhance the detection effect of small targets.

**Result:**

At last, model comparison experiments, ablation experiments, and daytime and nighttime pear detection experiments were carried out. In the model comparison experiments, the detection effect of YOLO-P was better than other lightweight networks. The results showed that the module’s average precision (AP) was 97.6%, which was 1.8% higher than the precision of the original YOLOv5s. The model volume had been compressed by 39.4%, from 13.7MB to only 8.3MB. Ablation experiments verified the effectiveness of the proposed method. In the daytime and nighttime pear detection experiments, an embedded industrial computer was used to test the performance of YOLO-P against backgrounds of different complexities and when fruits are in different degrees of shade.

**Discussion:**

The results showed that YOLO-P achieved the highest F1 score (96.1%) and frames per second (FPS) (32 FPS). It was sufficient for the picking robot to quickly and accurately detect pears in orchards. The proposed method can quickly and accurately detect pears in unstructured environments. YOLO-P provides support for automated pear picking and can be a reference for other types of fruit detection in similar environments.

## 1 Introduction

Pears are a common fruit which have rich nutrition and good taste. China grows the most pear trees, with a pear tree planting area that accounts for 67.30% of the global total pear tree planting area (Food and Agriculture Organization of the United Nations, 2022). However, the continuous loss of agricultural labor in recent years has led to a substantial increase in the cost of manual picking. The problem became more prominent after the COVID-19 pandemic (Nawaz et al., 2021). Therefore, efficient picking machines are a current research focus and an area of importance in orchard intelligence. Automated picking can increase the income of fruit farmers and promote economic development (Galvan et al., 2022).

Fruit detection is one of the most important steps for orchard picking robots working autonomously. At present, some scholars have used machine learning methods, especially based on color features, to detect fruits which are significantly different from the background color. For example, Si et al. (2010) proposed a method based on the red–green differential separation which used the contour formed by the shape of fruit to segment the red apple and green background. But this method is no longer effective when the target is similar to the background color, because some fruits (like some varieties of apples and mangoes) are green even when they are ripe. Xiang et al. (2012) used the curvature of overlapping tomato boundary lines to detect shaded tomatoes, but the accuracy for large shaded areas was only 76.9%. Compared with the deep learning technology that has developed rapidly in recent years, traditional machine learning methods exposed more limitations, such as low speed, low detection accuracy, and poor universality. Also, the designed algorithm can detect only a single target. As far as computers are concerned, the low-level features that machine learning uses are difficult to extract deep semantic information (Arrieta et al., 2020), making it unsuitable for online equipment and fruit detection in the complex and changeable environment of orchards.

Deep learning technology has been widely used in target detection in orchards. Object detection based on deep learning is mainly divided into a two-stage algorithm and a one-stage algorithm. Two-stage algorithms have been extensively studied due to high accuracy in the field of agriculture. Zhang et al. (2020) developed a detection system for apples and branches based on VGG-19 and Faster R-CNN for the vibration harvest. The mean average precision (mAP) for detecting apples was 82.4% and the fitting degree to the branches and trunks was over 90%. Tu et al. (2020) used a red, green, blue plus depth (RGB-D) camera to obtain the red, green, blue (RGB) image and depth information of passion fruit and combine them. A multi-scale-based Faster Region-based Convolutional Neural Network (R-CNN) network (MS-FRCNN) was proposed, which achieved an F1 score of 90.9%. Yan et al. (2019) improved the Region of interest (ROI) pooling layer of Faster R-CNN and combined VGG16 to detect 11 types of *Rosa roxbunghii* with different shapes; an average precision of 92.01% was obtained. The accuracy of two-stage detection is high. However, the huge number of parameters leads to increased computation costs and decreased detection speeds, which make it difficult to apply to online detection tasks.

The one-stage detection algorithm can greatly improve detection speed while maintaining detection accuracy because there is no process of generating candidate regions. Peng et al. (2018) used ResNet-101 to improve Single shot detector (SSD) for four kinds of fruit detection: citrus, apple, orange, and lychee. Compared with the original SSD, the average accuracy increased by 3.15%, and performance improved in shaded conditions. The “You Only Look Once” (Redmon et al., 2016; Redmon and Farhadi, 2017; Redmon and Farhadi, 2018; and Bochkovskiy et al., 2020) series of algorithms was born in 2015. This series has reached its fifth iteration and shows the trend and potential of continuous updating and strengthening. Due to the continuous integration of the latest network optimization tricks, both speed and accuracy can be maintained at a high level. The YOLO algorithm is considered to be one of the most successful one-stage detection networks. Bresilla et al., 2019 established an apple detection model based on YOLOv2. By adding computer-drawn images to assist training, the author found that synthesized images can reduce the position loss of the network and better locate the target. Pear detection was performed by transfer learning and the model achieved an F1 score of 0.87%. Liu et al. (2022) improved YOLOv3 to detect pineapples and calculated the 3D coordinates based on binocular vision cameras. The average precision (AP) value of fruit detection was 97.55% and the average relative error of binocular camera positioning was 24.4 mm. Xu et al. (2020) improved the backbone of YOLOv3, modified the batch normalization layer to group normalization, and used Soft-NMS to replace the original network management system (NMS) bounding box filter. The author proposed an image enhancement method to improve backlit images. The model finally got an F1 score of 97.7%. Parico and Ahamed (2021) improved YOLOv4, realizing fruit counting through a unique identity document (ID) method, which could meet the requirements of online operation. Zheng et al. (2022) used the improved YOLOv4 to detect tomatoes in a natural environment, and accuracy was improved by 1.52% compared with the original model. Jiang et al. (2022) integrated a non-local attention module and a convolutional block attention module (CBAM) into YOLOv4 to detect growing apples. Improved extraction ability of advanced features and perception of regions of interest. The test achieved an AP of 97.2%. Lu et al. (2022) used the improved YOLOv4 to calculate the number and the size of fruits on the whole apple tree. The network had the highest detection rate during fruit picking. This research enhanced the management ability of fruit trees. Zhang et al. (2022) proposed real-time strawberry detection network (RTSD-Net) by improving YOLOv4-tiny’s cross stage partial network (CSPNet). The detection of strawberries with the embedded system Jetson Nano had a detection speed of 25.2 FPS; hence, the real-time performance of the network was good. Chen et al. (2022) used YOLOv5 to detect citrus fruits and proposed a citrus ripeness detection algorithm that combined visual saliency with residual network (RESNet)-34. The accuracy of the model could reach 95.07%. Yan et al. (2021) used an improved YOLOv5 to detect apples and judge whether the fruit could be grasped by the picking machine. The model obtained a mAP of 86.75% and an F1 score of 87.49%. Yao et al. (2021) improved YOLOv5 by adding a small object detection layer, inserting a squeeze and excitation (SE) layer, and using a complete intersection over union (CIoU) loss function. The model achieved a mAP of 94.7% in an experiment detecting kiwifruit defects. Sozzi et al. (2022) utilized multiple networks to detect white grapes under different lighting conditions, against different backgrounds, and at different growth stages. The F1 score of YOLOv5x in the experiment was 0.76% and the detection speed was 31 FPS. Summarizing the above studies, using a one-stage algorithm such as YOLOv5 has become the most common method of fruit detection. However, the detection speed and accuracy of the network is still one of the problems to be solved urgently, and the existing research rarely considers the complex natural environment of the orchard.

YOLOv5 can achieve good results in datasets such as PASCAL VOC (Everingham et al., 2015) and COCO (Lin et al., 2014). However, for detection tasks in agriculture, the complete YOLOv5 network produces more performance redundancy. Even the light version of YOLOv5s struggles to achieve satisfactory results in orchards. At the same time, the background in orchards can be complex and fruits are easily shaded by other objects. The nighttime environment also has a significant impact on the effectiveness of detection. The existing YOLOv5 algorithm is facing great challenges, especially in low-performance devices, such as industrial computers, in online detection. Therefore, the purpose of this research was to design the YOLO-P network for fast and efficient detection of pears against complex backgrounds, in shade and during night picking. This method was based on YOLOv5. We designed a new module, named an inverted shuffle block, which can be applied in deeper layers to solve the problem of small targets missing in detection. We replaced some of the CBS structure in the YOLOv5 backbone with a shuffle block and an inverted shuffle block to form a new backbone. A CBAM was inserted into the new backbone to improve the ability to capture key features of pears. In addition, the activation functions in the remaining CBS of the entire network were replaced by Hard-Swish to improve the running speed. The detection effect of this method had been verified under different degrees of shade and background complexity during daytime and nighttime. YOLO-P can be used for fast and accurate detection of pears in orchards and can a references for other types of fruit detection in similar environments.

## 2 Pear detection framework

**Figure 1:**
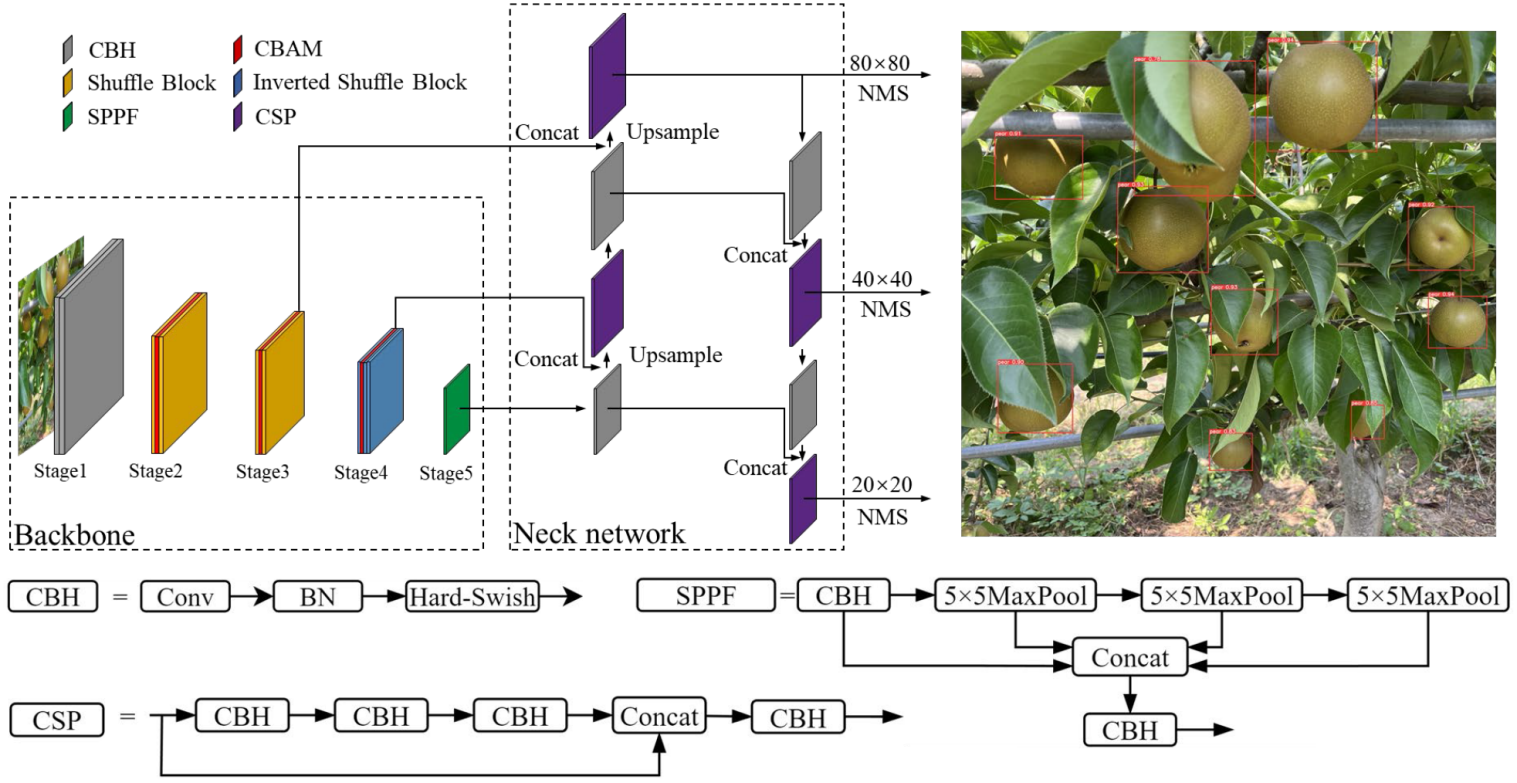
The network structure of YOLO-P.

As one of the most mature, stable, and effective target detection algorithms currently available, YOLOv5 consists of three main parts: a backbone network, neck network, and classifier. The backbone is cross stage partial (CSP)-DarkNet53, which is used to extract different scale feature information from images. The neck network is path aggregation network (PANet) (Liu et al., 2018) with feature pyramid network (FPN), which is used to fuse feature information. The classifier outputs bounding boxes of large, medium, and small scales to complete the target detection. The YOLO-P method proposed in this paper is based on YOLOv5 and the structure is shown in **Figure 1**. The CBS structure in the second and third stages of the YOLOv5 backbone were replaced with a shuffle block. An inverted shuffle block was designed and used to replace the CBS structure of the fourth stage. This new backbone could extract features of distant pears in images more efficiently. CBAM was inserted in the new backbone to improve the important information perception capability of pears. The sigmoid linear unit (SiLU) activation function in the rest of the CBS structure was replaced with Hard-Swish to improve the running speed of the network. A weighted confidence loss function was designed to strengthen the detection effect of small targets. The details of the improvements are described below.

### 2.1 Backbone network

**Figure 2:**
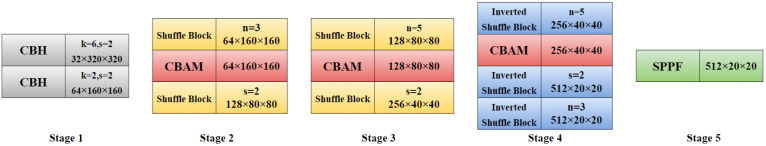
YOLO-P’s backbone. k is convolutional kernel size, s is stride, and n is the number of module’s repetitions. Unspecified k is 3, s is 1, and n is 1.

Ma et al. (2018) proposed that making the input and output feature maps equal, reducing convolution and element-wise operations, and integrating the network structure would help improve the inference speed of the network. Tan and Le (2020) suggested that increasing the depth of the network could result in richer features but may cause gradients to disappear. Increasing the width of the network results in finer-grained features, but it may fail to learn deep features. Therefore, it is necessary to balance the depth and width of the network to achieve the best results. **Figure 2** shows the backbone of YOLO-P, built following the above lightweight network design principles, and lists the size of the output feature map (C×H×W). The input image size of the network is 3 × 640 × 640. The first stage is downsampling through two convolutional layers to obtain a feature map with a size of 64 × 160 × 160. The second and third stages use the shuffle block to extract features in the middle and shallow layers and downsample twice to obtain a feature map with a size of 256 × 40 × 40. The fourth stage uses the inverted shuffle block to extract features in deeper layers of the network and downsamples to obtain a 512 × 20 ×20 feature map. The fifth stage uses the improved spatial pyramid polling (SPPF) module in the deepest layer of the network to fuse the receptive field information of different scales. Finally, the SPPF output of the fifth stage and the output after the third and fourth stages’ CBAMs are sent to the neck network of YOLO-P.

#### 2.1.1 Feature extraction

**Figure 3:**
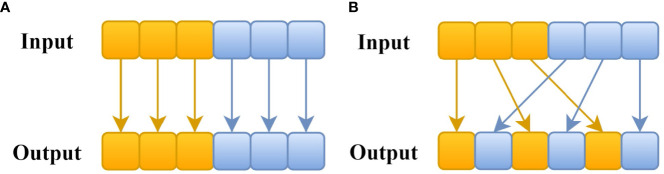
**(A)** Group Convolution; **(B)** Group Convolution with Channel Shuffle.

The CSP-DarkNet53 of the YOLOv5 backbone uses a large number of CBS (Conv, Batch Norm, SiLU) structures which are suitable for target detection of complex features. However, this combination occupies a large amount of computation, and it is difficult for the application to run online in embedded devices. Therefore, this part needed to be optimized first. Xie et al. (2017) proposed the concept of group convolution in ResNeXt, which can effectively reduce the computational load of the network, as shown in **Figure 3A**. But there was no information exchange between groups and reduced the feature extraction ability. Based on the idea of group convolution, Ma et al. (2018) proposed a lightweight neural network ShuffleNetv2 that added channel shuffle in shuffle block. **Figure 3B** shows the group convolution process with channel shuffle. The channels between groups are shuffled before output. The resulting information exchange enables feature extraction to be done more efficiently.

##### 2.1.1.1 Shuffle block

**Figure 4:**
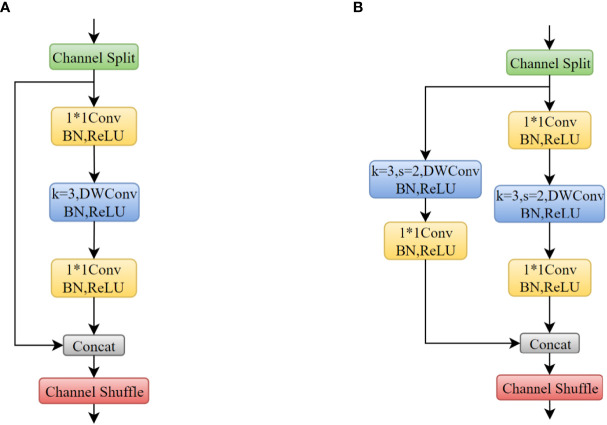
**(A)** Shuffle Block (s=1); **(B)** Shuffle Block (s=2). a * b means the width and height of the convolution kernel.

The shuffle block includes two cases where the stride is 1 and 2, respectively, as shown in **Figure 4**. First, the input feature matrix channels was divided into two groups by channel split and pass through two branches. If stride was 1, a residual structure containing 1×1Conv, 3×3DwConv and 1×1Conv in one branch was performed. If stride was 2 (downsampling), an additional 3×3DwConv and a 1×1Conv on the other branch was performed. The two branches were concatenated and the feature map was outputted through channel shuffle.

##### 2.1.1.2 Inverted shuffle block

**Figure 5:**
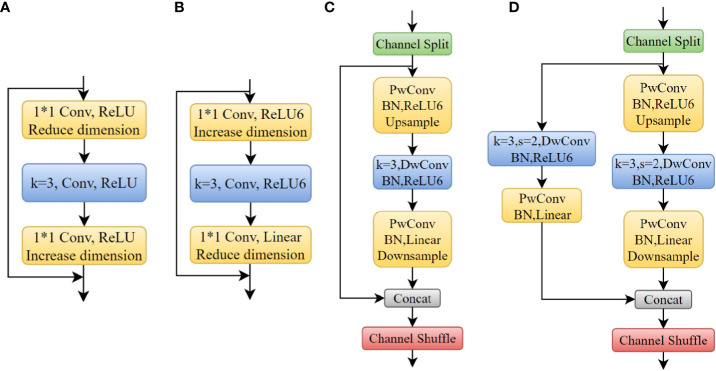
**(A)** Residual Block; **(B)** Inverted Residual Block; **(C)** Inverted Shuffle Block (s=1); **(D)** Inverted Shuffle Block (s=2). a * b means the width and height of the convolution kernel.

The residual structure in CSP-DarkNet53 is shown in **Figure 5A**. First, increases the dimension of the feature map increased and the dimension was reduced to extract features. However, there could be more zeros in the convolution kernel’s parameter of deeper layers. Directly increasing dimension brings difficulties to deep layers’ feature extraction. In MobileNet (Howard et al., 2017), an inverted residual structure that first reduced the dimension of the feature map and then increased the dimension was proposed to extract more information, as shown in **Figure 5B**. Inspired by lightweight networks such as ShuffleNet and MobileNet, this study designed the inverted shuffle block used in deeper layers of network (the fourth stage of backbone), as shown in **Figures 5C, D**. The reversed structure made it easier to extract features from small objects. It was similar to shuffle block, but the residual structure of the branch was changed to an inverted residual structure. Similarly, if the stride was 2 (downsampling), an additional 3×3DwConv and a PwConv on the branch of the inverted residual structure was performed. The two branches were concatenated together and output the feature map was outputted through channel shuffle.

#### 2.1.2 Attention module

**Figure 6:**
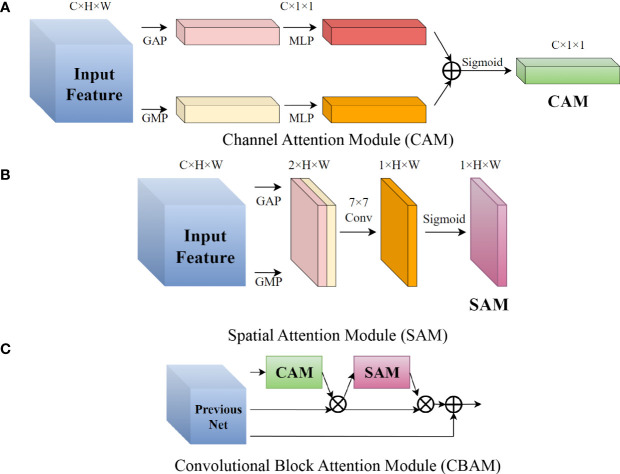
Schematic diagram of the CBAM structure in YOLO-P. **(A)** Channel Attention Module (CAM) **(B)** Spatial Attention Module (SAM) **(C)** Convolutional Block Attention Module (CBAM).

Attention mechanism is a way to reinforce important information and suppress secondary information in a neural network. Application in the field of image object detection had proved attention mechanism’s effectiveness. The CBAM is a lightweight soft attention module that is divided into channel and spatial parts (Woo et al., 2018). The channel attention module (CAM) when the inputs were C × H × W is shown in **Figure 6A**. We then performed global average pooling (GAP) and global maximum pooling (GMP) to the feature map in order to obtain two C × 1 × 1 feature matrices and send them to a multi-layer perceptron which has two layers. This was then summed and activated to get the channel attention vector. CAM focuses on what is in the feature map. The Spatial Attention Module (SAM) is shown in **Figure 6B**; we then performed GAP and GMP on the channel dimensions of the feature map to obtain a 2 × H × W feature matrix, then a 7 × 7 convolutional layer and activation to get a 1 × H × W spatial attention vector. The purpose of SAM is to more prominently express the characteristics of key regions. Each pixel of the feature map generates a weighted mask and outputs it, which reinforces where the key target is. **Figure 6C** shows CBAM. The channel attention vector obtained by CAM was first multiplied with input feature map. Then the resulting feature map was multiplied by spatial attention matrix obtained by SAM. Finally, the output of CBAM is obtained through the residual structure. The sequence of using CAM and then SAM to correct the feature maps was based on the characteristics of the human cerebral cortex, Woo et al. (2018) experiments also verified this. We applied CBAM to the second, third, and fourth stages of YOLO-P’s backbone. Following experiments by Park et al. (2018), we inserted the attention module at the bottleneck of the network, i.e., before the downsampling layer. We then connected the output of CBAM to the neck network of YOLO-P for better feature fusion.

### 2.2 Activation function

The activation function of the network was mainly improved in two aspects. First was to replace the SiLU activation function for all CBS structures in YOLOv5 with Hard-Swish, and the second was to use the linear activation function for the last convolution layer in the inverted shuffle block.

First, all CBS structures in YOLOv5 used SiLU as an activation function. For the network applied to embedded devices, obviously the linear activation function could make the network faster. Hard-Swish (Howard et al., 2019) activation function was bounded up and down. The non-monotonic and piecewise linear characteristics reduced the amount of calculation. It was beneficial to eliminate saturation and make the feature expression ability better. All Conv, Batch Norm, Hard-swish (CBH) structures in YOLO-P’s backbone and neck network used Hard-Swish as an activation function. Equation (1) is the Hard-Swish expression where *x_in_* represents the input of the activation function. Second, ReLU was used as an activation function after most convolutional layers in the original shuffle block. However, due to the inverted residual structure of the inverted shuffle block, first an increase in dimension and then a reduction in dimension made the final output a low-dimensional feature vector. Although ReLU can better express high-dimensional features, it has serious loss of low-dimensional feature information (Sandler et al., 2018). In order to ensure the feature information was not lost and to better match the complete output of the inverted residual, each branch of the last convolutional layer of inverted shuffle block’s used a linear activation function.


(1) $$\text{Hard-Swish(}x_{in}\text{)=}x_{in}\frac{\text{ReLU6(}x_{in}+3)}{6}$$

(2) $$\text{ReLU6(}x_{in}\text{)=}\min(\max(x_{in},0),6)$$

### 2.3 Loss function

Since the detection target type of the model was only pear, we did not set the class loss. The loss function of YOLO-P consists of confidence loss and location loss. Equation 3 shows confidence loss which was used to measure the probability that the predicted bounding box contained the real target. It was calculated by using binary cross entropy (BCE). In In Equations 3 and 4, *I* is the intersection area of the ground-truth box and predicted bounding box, *U* is the area of the union, *C_i_* is the prediction confidence, *N* is the total number of samples, and *spl* represents all samples. According to the structure of the YOLO-P predictor, different weights *K*_1_, *K*_2_, and *K*_3_ are adopted on the three prediction layers of small, medium, and large to strengthen the targets’ detection ability of different scales. The confidence loss is shown in Equation 5. Since pears with a greater distance (small objects on the image) are more difficult to detect, we took *K*_1_, *K*_2,_ and *K*_3_ as 6.0, 1.0, and 0.5 in YOLO-P, respectively.


(3) $$L_{conf}^{'}=-\frac{\sum_{i\in spl}(\frac{I}{U}\ln(C_{i}^{'})+(1-\frac{I}{U})\ln(1-C_{i}^{'}))}{N}$$

(4) $$C_{i}^{'}=\text{sigmoid(}C_{i})$$

(5) $$L_{conf}=6.0\cdot L_{conf}^{small}+1.0\cdot L_{conf}^{medium}+0.5\cdot L_{conf}^{large}$$

The location loss measures the location error between predicted bounding box and ground-truth box. Zheng et al. (2020) pointed out that the regression loss of bounding box should take the overlapping area, the distance between center points of the box, and the aspect ratio into account. In this study, we used CIoU loss as the location loss of YOLO-P, as shown in Equations 6–8, where *w_gt_* and *b_gt_* are the length and width of ground-truth box, *w_p_* and *b_p_* are the length and width of the predicted bounding box, *d* is the Euclidean distance between the predicted box and the ground-truth box, and c is the diagonal distance of the union of the predicted box and the ground-truth box. The CIoU loss can directly minimize the distance between two boxes (Zheng et al., 2020), so it has a faster convergence rate.


(6) $$L_{loc}=1-(\frac{I}{U}-(\frac{d^{2}}{c^{2}}+\alpha v))$$

(7) $$\alpha =\frac{v}{(1-\frac{I}{U})+v}$$

(8) $$v=\frac{4}{\pi }{\left(\arctan\frac{w_{gt}}{b_{gt}}-\text{arc}tan\frac{w_{p}}{b_{p}}\right)}^{2}$$

Combined with confidence loss and location loss, the loss function of YOLO-P is shown in Equation 9.


(9) $$Loss=L_{conf}+L_{loc}$$

## 3 Experiments

### 3.1 Dataset

Images required for the experiment were collected at a pear planting base located in Gaochun District, Nanjing City, Jiangsu Province, China. In this research, Akidzuki pears were used as detection targets. In August 2022, images were captured using a Sony FDR-AX60 4K camera with a sensor type of 1/2.5 stacked complementary metal-oxide-semiconductor (CMOS), and a total of 533 images containing Akidzuki pears were captured as training samples while 118 images different from the training samples were taken for model testing. In addition to normal daytime lighting, the dataset also contained samples at night. The images at night were taken with the aid of a 1000 lm light source. Images contained shaded pears and complex backgrounds. We used ImageLabel to annotate images and perform data augmentation by randomly selecting three of the following augmentation strategies: (1) 50% probability of horizontal mirror flip, (2) 50% probability of vertical mirror flip, (3) random scaling 80–95%, (4) random brightness adjustment to 35–150%, (5) randomly added Gaussian blur, or (6) randomly added Gaussian noise. The images that could not be used for training were eliminated, and the training dataset was finally expanded to 5257 images. The expanded image inherited the previous annotations with 55496 labels in total. According to the ratio of 8 : 2, the dataset was divided into a training set and a validation set, which had 4206 and 1051 images, respectively. All images were stored in JPG format. The details of the dataset are shown in **Table 1**.

**Table 1:** Details of the pear image dataset.

	Uncomplicated background	Moderately complex background	Extremely complex background	Daytime	Nighttime	Total images
Number of images	1209	1630	2418	3680	1577	5257

The difference in the distance between the camera and the pear will result in different scales of the collected images. The further the distance, the smaller the target. At this time, most areas of the image will be covered by useless background and increase the image’s background complexity. The disordered background in the orchard makes it more challenging for the model to detect objects. Also, the number of smaller objects will increase significantly. According to the distance between the camera and the fruit, we divided the background of the image into three cases: uncomplicated, moderately complicated, and extremely complicated. Among them, the distance of 0.3–0.5 m was set for uncomplicated, while 0.5–1 m for moderately complicated, and farther than 1m for extremely complicated.

The pears on the fruit trees photographed by camera were sometimes shaded by leaves or other objects, and there were also cases where the pears might be shaded by each other. The shaded target would bring difficulties to detection. In order to specifically verify the reliability of YOLO-P in detecting such targets, we proposed a method for calculating the pears’ shaded degree. *K*_s_ was used to evaluate the degree of shade, which was the ratio of the shaded area to the total area of the pear in images. According to our previous experiments, it was extremely difficult to detect when *Ks* was higher than 0.6, so only the case of *K*_s_*<* 0.6 was considered in this study, as shown in **Table 2**.

**Table 2:** Index of shaded pear’s degree in the dataset.

	Evaluation indicators
Not shaded or slightly shaded	0≤*K*_s_*≤*0.2
Medium shaded	0.2<*K*_s_*≤*0.4
Serious shaded	0.4<*K*_s_*≤*0.6

### 3.2 Experimental environment and parameters

Training of YOLO-P was carried out in a Windows 10 environment. The graphics processing unit (GPU) was Nvidia GeForce RTX 3060, the central processing unit (CPU) was AMD Ryzen 7 5800, and the memory was 32 GB. We used the Pytorch1.8.1 framework, CUDA 11.1 computing platform and CUDNN 8.1 deep neural network acceleration library.

The momentum decay and weight decay of all models during training were designed to be 0.9 and 0.0005, respectively, and the initial learning rate was 0.01. At the same time, the cosine annealing algorithm was used to optimize the learning rate. We used three rounds of epoch to warmup in order to stabilize the early training model. The warmup momentum was 0.8 and the batch size was set to 32. We used Adam as the optimizer with 500 training epochs. To prevent overfitting, the model would automatically stop training if there was no accuracy improvement in the last 50 training epochs.

### 3.3 Evaluation indicators

A variety of indicators could be used to evaluate the quality of the model in different experimental contexts, such as precision (P), recall (R), F1 score, AP, mAP, FPS, FLOPs, model volume, etc. The higher the P, R, F1 score, and AP, the more reliable the model would be. Their computation consists of true positives (TP), false positives (FP), and false negatives (FN), as shown in Equations 10-13 respectively. The intersection over union (IoU) threshold in AP took 0.5 (AP@0.5). It is worth mentioning that there was only one category of pears in this study, so AP and mAP were equal.


(10) $$P=\frac{TP}{TP+FP}$$

(11) $$R=\frac{TP}{TP+FN}$$

(12) $$F1=\frac{2PR}{P+R}$$

(13) $$AP=\int_{0}^{1}P(R)\text{d}R$$

Model volume refers to the size of weight file obtained after training. FPS refers to the number of images the model can process per second. FLOPs is the total floating-point operations of the model, as shown in Equation (14), where *N* represents all convolutional layers, *L_i_* and *C_i_* are the output feature layer size and number of channels of the current layer, respectively, *K_i_* is the number of convolution kernels of the current layer, and *C_i-_*_1_ is the number of input channels of the current layer. Like the model volume, the higher the FLOPs and the more complex the model, the slower the operation speed and the lower the FPS.


(14) $$\text{FLOPs=}\sum_{i\in [1,N]}L_{i}^{2}\times K_{i}^{2}\times C_{i}\times C_{i-1}$$

### 3.4 Experiments results

#### 3.4.1 Model comparison experiments

Since YOLO-P is a one-stage model, the purpose is to run at high speed on low-performance devices, so it is not meaningful to compare with the two-stage model. We selected several mainstream lightweight networks including RegNet, MobileNetv3, and EfficientNetv2 to compare with YOLO-P. RegNet (Radosavovic et al., 2020) optimized design space of the network to obtain optimal solution. MobileNetv3 (Howard et al., 2019) added squeeze excitation attention to the inverted residual module, and reduced the amount of computation without losing accuracy by improving the structure of the last stage. EfficientNetv2 (Tan and Le, 2021) improved feature extraction efficiency by introducing Fused-MBConv. In order to make the model volume more similar to YOLO-P, we replaced the backbone of YOLOv5s with the above three networks. At the same time, the classic YOLOv5s model was used for comparison. In the model comparison experiments of this section, we selected P, AP@0.5, FLOPs, and module volume as evaluation indicators. The test results are shown in **Table 3**.

**Table 3:** Results of model comparison experiments.

	Precision (%)	AP@0.5 (%)	FLOPs (G)	Model Volume (MB)
RegNet-YOLO	92.8	90.3	13.4	14.6
MobileNet-YOLO	95.4	95.2	**7.3**	9.2
EffiecientNet-YOLO	95.6	95.0	14.4	17.8
YOLOv5s	96.0	95.8	15.9	13.7
YOLO-P	**98.1**	**97.6**	10.1	**8.3**

Bold means the best score achieved in that category.

From the data in **Table 3**, it can be seen that YOLO-P achieved the best AP in section’s experiments, which was 97.6% and it was 1.8% higher than its original network. RegNet-YOLO had the lowest AP. Although the FLOPs of YOLO-P was not the lowest, we got the smallest model volume which was only 8.3 MB. Compared with YOLOv5s, it was 39.4% smaller. MobileNet-YOLO had the lowest FLOPs of only 7.3 G, which is related to the reduction of last stage in this network. Model comparison experiments showed that the combination of shuffle block and inverted shuffle block was reliable. The proposed YOLO-P model could detect pears in orchards with a smaller model volume and high accuracy.

#### 3.4.2 Ablation experiments

We conducted ablation experiments on YOLO-P and discussed the performance improvement of YOLOv5s with new modules and new structures. New operations included shuffle block, inverted shuffle block, Hard-Swish activation function used in CBH, and inserted CBAM. We designed four sets of experiments in this section. In the T1 experiment, the four CBS groups and their corresponding downsampling modules in the YOLOv5s backbone network were replaced with shuffle blocks. In the T2 experiment, the four CBS groups and their corresponding downsampling modules in the YOLOv5s backbone network were replaced with an inverted shuffle block. The number of module repetitions in both T1 and T2 was the same as YOLO-P. In the T3 experiment, all four CBS groups were replaced with the same shuffle block and inverted shuffle block as YOLO-P. The T4 experiment used Hard-Swish on the basis of the T3. Finally, full YOLO-P network was CBAM’s insertion. In the model ablation experiments of this section, we selected precision, AP0.5and FLOPs as evaluation indicators: the test results are shown in **Table 4**.

**Table 4:** Results of ablation experiments.

	Shuffle Block	Inverted Shuffle Block	Hard-Swish	CBAM	Precision (%)	AP@0.5 (%)	FLOPs (G)
YOLOv5s					96.0	95.8	15.9
T1	√				94.3	93.9	10.6
T2		√			94.8	94.7	**9.3**
T3	√	√			96.2	95.9	10.0
T4	√	√	√		96.9	96.5	10.0
YOLO-P	√	√	√	√	**98.1**	**97.6**	10.1

Bold means the best score achieved in that category.

It can be seen from **Table 4** that only using a shuffle block or an inverted shuffle block in the backbone was not as good as the AP obtained by YOLOv5s, because the inverted structure is not suitable for shallow networks. Also, the use of upsampling in deep networks reduced the ability to detect small objects. We used different structures in shallow and deep layers of the network to deal with different sized targets. It would be easier to detect targets with inconspicuous feature expressions by combining the characteristics and advantages of the two modules. The AP obtained by the T3 experiment was similar to original network, which was only 0.1% higher than YOLOv5s. However, due to the influence of the channel shuffle, the calculation amount of model was reduced which made the FLOPs reduce, and the detection speed was also be improved. The model’s AP was improved by 0.6% after optimizing the SiLU activation function to Hard-Swish. On this basis, the feature extraction ability was further strengthened by inserting CBAM, which made AP increase by 1.1%, reaching 97.6%. The comparison of four sets of experiments above proved that the proposed improved application is feasible in the pear detection network.

#### 3.4.3 Pear detection experiments

**Figure 7:**
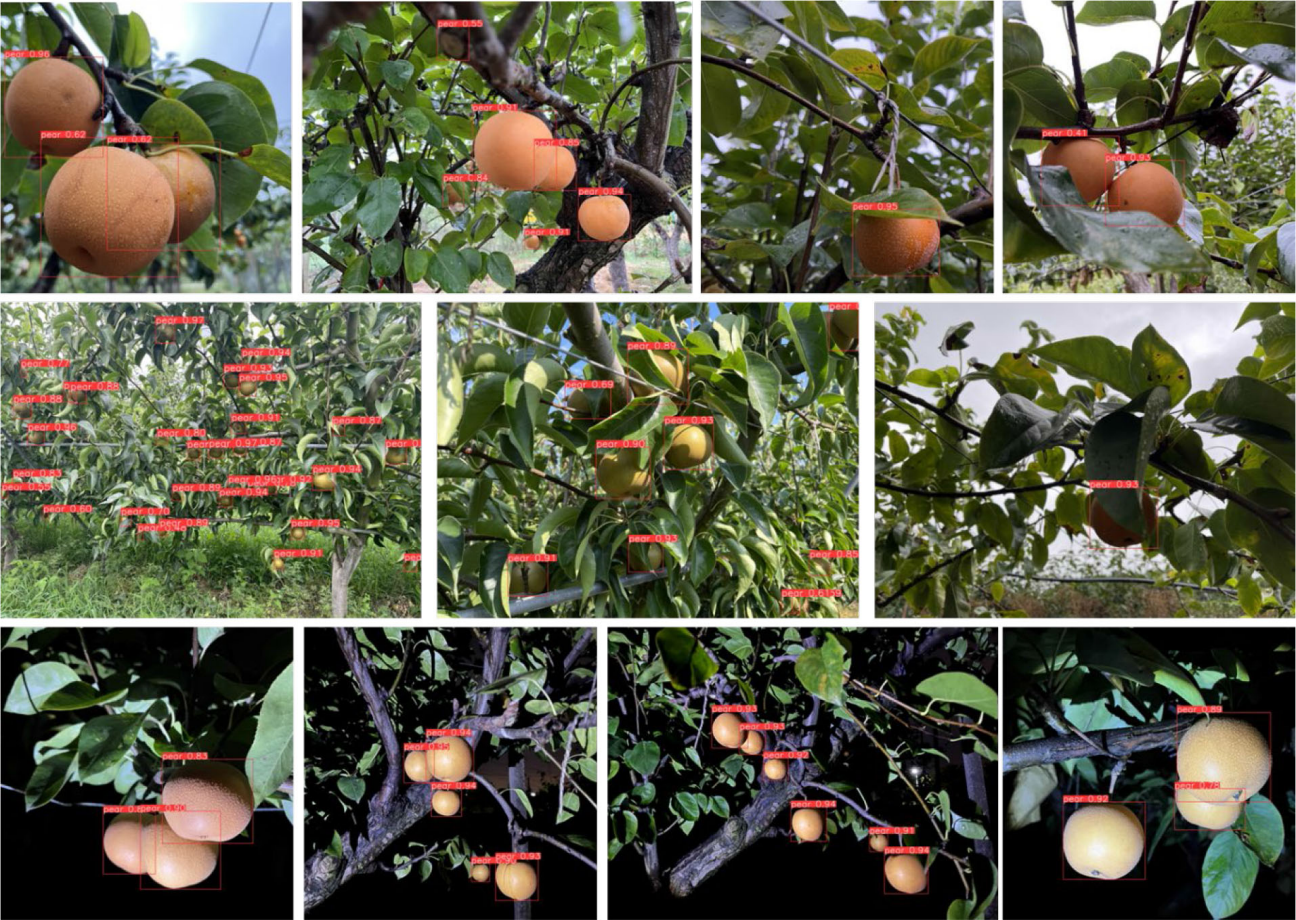
The detecting effect of Akidzuki pear in complex environment.

Pear detection experiments were carried out on an industrial computer with limited computing resources in order to verify the feasibility of YOLO-P online work. We chose the embedded industrial computer of model DTB-3049-H310 produced by Dongtintech. The operating environment was Ubuntu 18.04, CPU was i7 9700 with 16 GB memory and it was without GPU. Detection experiments considered many situations of an intelligent picking robot in orchard. Different types of picking machinery working at different distances resulted in different degrees of background complexity. Dense foliage made pears shaded. For efficiency purposes, picking should be done not only during the daytime, but also at night. The experiment used 59 daytime and 57 nighttime pear images that different from the training samples, with a total of 649 labels. Three models (YOLOv5s, MobileNet-YOLO, YOLO-P) were selected in this section’s experiments. The models’ detection abilities under different background complexities and different degrees of shaded were respectively studied. We set the confidence threshold of the detection model to 0.4, i.e., confidence below 0.4 was not annotated in the image. The P, R, and F1 score were calculated by counting TP, FP and FN. FPS of the model operation were recorded. The overall test results are shown in **Table 5**. Pears that were detected by YOLO-P are shown in **Figure 7**.

**Table 5:** Result of Akidzuki pear detection experiments.

	Precision (%)	F1 (%)	FPS
MobileNet-YOLO	90.1	89.6	28
YOLOv5s	94.8	92.8	19
YOLO-P	**97.3**	**96.1**	**32**

Bold means the best score achieved in that category.

##### 3.4.3.1 Experiments during daytime

There was sufficient sunlight during the daytime: pears were easily detected when the background was not complicated (the target was obvious) and the degree of shade was low. However, the shade led to reduction of features or the image taken from a long distance led to fewer pixels on the target which would weaken the feature representation of pears. In this section, detection experiments were carried out on pears in different situations according to the proposed method of calculating background complexity and shaded degree under sufficient light during daytime.

**Figure 8:**
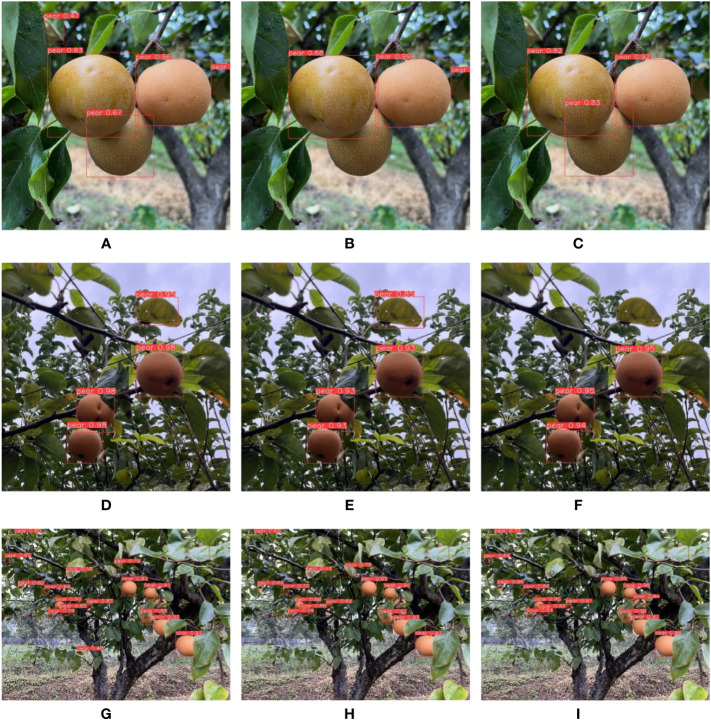
From left to right are the detection effects of YOLOv5s, MobileNet-YOLO and YOLO-P. **(A–C)** Uncomplicated background; **(D–F)** Moderately complex background; **(G–I)** Extremely complex background.

First, experiments of different background complexities were carried out. We measured the background complexity by the distance between camera and pears. The F1 score obtained in this section is shown in **Table 6**. The experiments images are shown in **Figure 8**. **Figures 8A–C** are images of pears in uncomplicated backgrounds. YOLO-P detected all objects accurately. There were two false detections in YOLOv5s. MobileNet-YOLO did not detect a pear that had been shaded below. **Figures 8D–F** are images of pears in moderately complex backgrounds. All three networks detected all targets, but both YOLOv5s and MobileNet-YOLO mistakenly marked a dead leaf as a pear. **Figures 8G–I** are images of pears in extremely complex backgrounds. The environment of these images was relatively harsh. There were 15 valid targets in the image and many pears were seriously shaded. MobileNet-YOLO missed four targets. YOLOv5s and YOLO-P both missed two targets, but YOLOv5s had two false detections. It can be seen from the experiment in this section that YOLO-P had strong anti-interference ability. Although YOLOv5s could also detect targets accurately, it often misidentified other objects such as dead leaves as pears due to similar features. Even in the case of extremely complex backgrounds and few pixels, YOLO-P hardly had false detections and missed detections.

**Table 6:** F1 score (%) in different background complexities experiments during daytime.

	Uncomplicated background	Moderately complex background	Extremely complex background	Average
YOLOv5s	95.5	95.1	93.2	94.6
MobileNet-YOLO	92.5	91.8	89.5	91.3
YOLO-P	**96.9**	**96.6**	**95.5**	**96.3**

Bold means the best score achieved in that category.

**Figure 9:**
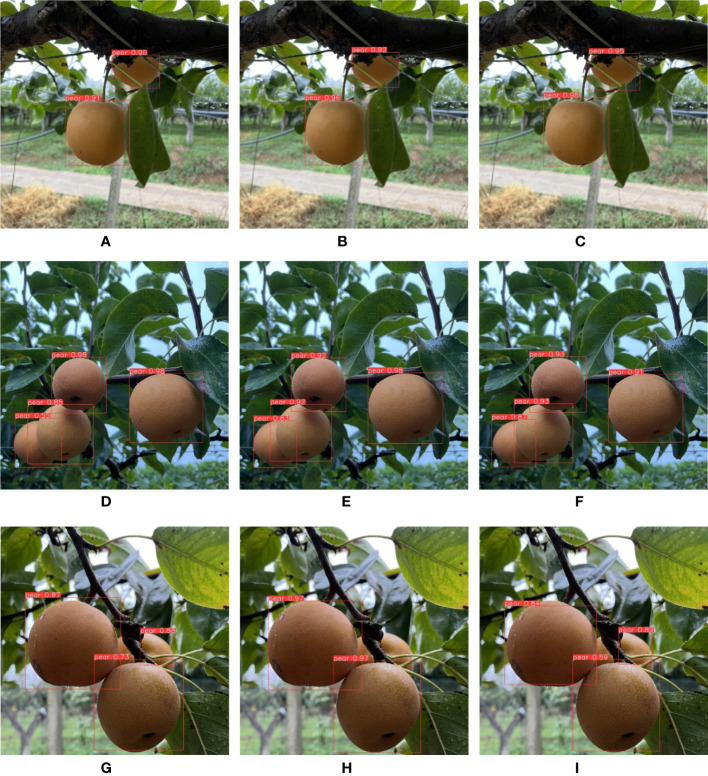
From left to right are the detection effects of YOLOv5s, MobileNet-YOLO and YOLO-P. **(A–C)** No shaded or slightly shaded; **(D–F)** Medium shaded; **(G–I)** Serious shaded.

In the experiment of different degrees of shade, the degree was measured by the shaded area of pears. The more severely shaded, the more difficult feature expression of pears in the image, and the more difficult to it was detect accurately. The F1 score obtained in this section is shown in **Table 7**. The experimental images are shown in **Figure 9**. **Figures 9A–C** are not shaded or slightly shaded pear images and **Figures 9D–F** are medium-shaded pear images. As can be seen from the figure, all three networks could detect the shaded pears, but YOLO-P always had the highest confidence in detecting shaded targets. **Figures 9G–I** are serious-shaded pear images. Only MobileNet-YOLO failed to detect serious shaded objects. YOLO-P was more stable against shade problems during the day due to its higher confidence.

**Table 7:** F1 score (%) in different shaded degrees experiments during daytime.

	Not shaded or slightly shaded	Medium shaded	Serious shaded	Average
YOLOv5s	94.8	94.3	94.2	94.4
MobileNet-YOLO	94.5	93.4	90.7	92.9
YOLO-P	**97.2**	**96.6**	**96.4**	**96.7**

Bold means the best score achieved in that category.

##### 3.4.3.2 Experiments during nighttime

The problem of nighttime detection is the presence of shadows. Shadows are very similar in color to the background, so shadows can also be considered as a form of detection. Shadows may have pixel values very similar to the external environment due to the uncertain lighting direction. The boundaries between the outline of pear and the environment become blurred. Therefore, detecting pears at night will be more difficult than during the day. In this section, detecting experiments were carried out under the illumination of an auxiliary light source at night.

**Figure 10:**
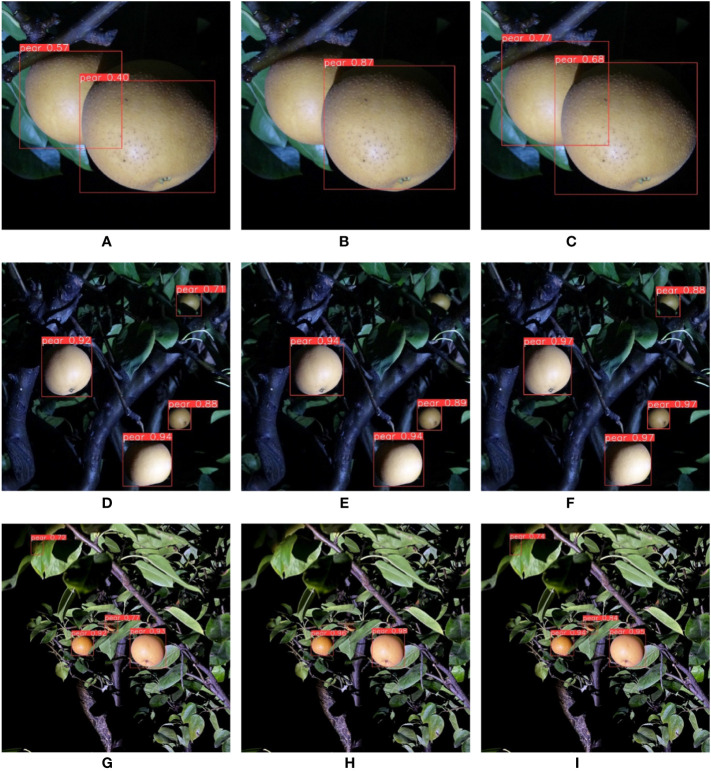
From left to right are the detection effects of YOLOv5s, MobileNet-YOLO and YOLO-P. **(A–C)** Uncomplicated background; **(D–F)** Moderately complex background; **(G–I)** Extremely complex background.

The F1 scores obtained by the experiments of different background complexity at night are shown in **Table 8**. The experiment images are shown in **Figure 10**. **Figures 10A–C** are images of pears in an uncomplicated background. It can be seen from the figure that MobileNet-YOLO missed a target. Both YOLOv5s and YOLO-P detected each objects successfully. But YOLOv5s had lower confidence and the location of the bounding box was not accurate. **Figures 10D–F** are images of pears in moderately complex backgrounds. The situation was similar to the previous group; although both YOLOv5s and YOLO-P detected all targets, YOLO-P had significantly higher confidence. **Figures 10G–I** are images of pears in extremely complex background. Both YOLOv5s and YOLO-P had a false detection, but they all detected a target in the middle of the image which was interfered with by a more complex shadow, while MobileNet-YOLO did not detect this target. The unclear edge of pears caused by nighttime illumination is one of the important reasons that affect the stability of the model. It can be concluded from the experiments that the performance of YOLO-P is better than other models in the complex background situation at night.

**Table 8:** F1 score (%) in different background complexities experiments during nighttime.

	Uncomplicated background	Moderately complex background	Extremely complex background	Average
YOLOv5s	92.8	92.5	88.9	91.4
MobileNet-YOLO	87.3	86.8	86.4	86.8
YOLO-P	**97.8**	**95.6**	**93.9**	**95.8**

Bold means the best score achieved in that category.

**Figure 11:**
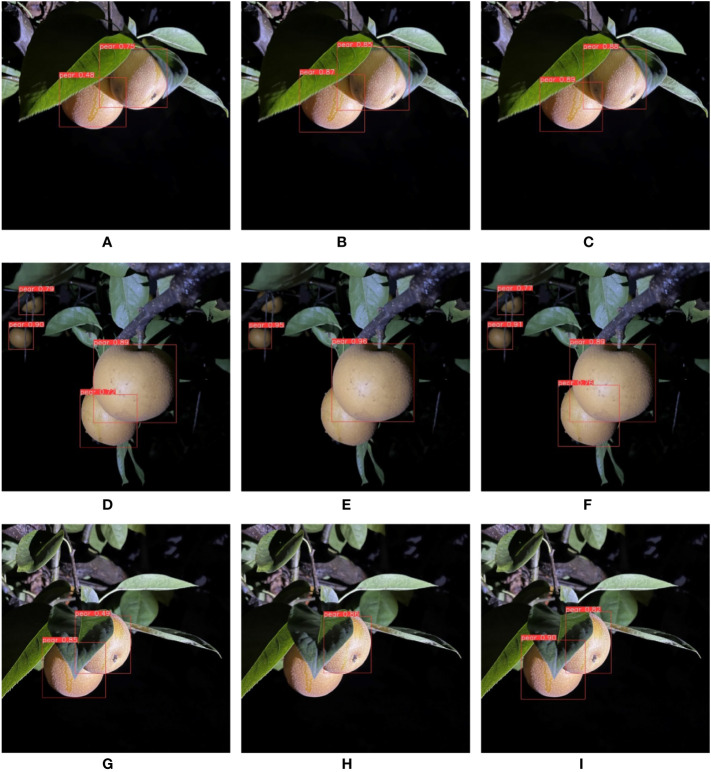
From left to right are the detection effects of YOLOv5s, MobileNet-YOLO and YOLO-P. **(A–C)** No shaded or slightly shaded; **(D–F)** Medium shaded; **(G–I)** Serious shaded.

The F1 scores obtained by the experiments of different shade degrees at night are shown in **Table 9**. The experiment images of at night are shown in **Figure 11**. **Figures 11A–C** are not shaded or slightly shaded pear images. All three networks detected the target accurately. **Figures 11D–F** are medium-shaded pear images. YOLOv5s and YOLO-P detected all targets. Neither of the two shaded fruits was successfully detected by MobileNet-YOLO. **Figures 11G–I** are serious-shaded pear images. YOLOv5s and YOLO-P detected all pears. But MobileNet-YOLO only detected one of the two targets. Likewise, YOLO-P had the highest confidence in this section’s experiment.

**Table 9:** F1 score (%) in different shaded degrees experiments during nighttime.

	Not shaded or slightly shaded	Medium shaded	Serious shaded	Average
YOLOv5s	91.5	90.6	90.2	90.8
MobileNet-YOLO	89.2	86.9	85.7	87.3
YOLO-P	**95.7**	**95.6**	**95.1**	**95.5**

Bold means the best score achieved in that category.

It can be seen that YOLO-P could accurately detect pears in various situations according to the above experiments. Although YOLOv5s could also accurately detect most targets, there were many false detections and lower confidence. Another weakness is that YOLOv5s needs more computing resources. MobileNet-YOLO was difficult to extract high-semantic features due to the insufficient feature extraction ability. Therefore, there was a high degree of missed detection which is especially evident in the case of high complexity and seriously shaded. In summary, YOLO-P had the best reliability in detecting pears in complex environments. YOLO-P had the best reliability in detecting pears under complex environments.

## 4 Discussion

Extensive research work has proved that building more complex datasets is the key to further improving the accuracy and robustness of deep learning models. For the automatic picking work in orchards, there are different shade patterns and backgrounds for each step the robot moves. Therefore, the scene it sees is far more complex than the images used for training. Although we collected as many complex images as possible, the variety of shaded fruits is too numerous. If a similar pattern of shaded fruits is not trained, the model will most likely be unable to recognize this object (although it looks remarkably easy to recognize). In this study, only the case where the fruit was shaded below 60% was considered. More diverse image data should be obtained in future work to deal with the more severely shaded fruit detection.

In experiments at night, we found that pixels in shadow-covered locations might be very similar to the outside environment, especially when the angle of the light source to the target was uncertain. This is one of the most important barriers to detecting pears at night. At present, some studies (Xu et al., 2020; Wang et al., 2022) have proved that the use of image enhancement can improve the accuracy of deep learning in harsh environments, especially in low light. If the models use some kind of machine learning method to preprocess the image and enhance the target boundary then input to neural network for recognition, the night detection ability of the model could be further improved.

Furthermore, only the detection of fully ripe pears was investigated in this study. In practice, picking in orchards should be done in batches. There may be cases that some pears are mature and some are not. Therefore, the intelligent detection of fruit ripeness is also one of the main research directions. Fruit ripeness can be judged by directly detecting the appearance characteristics (Chen et al., 2022). In addition, remote sensing can also be used for detection. From a macro perspective, the leaves of pear trees will become darker during the ripening season, and the fruits on pear trees may also have different characteristics. Remote sensing detection combined with deep learning may better judge fruit ripeness, thereby helping intelligent picking in orchards.

## 5 Conclusions

The cost of manual picking has gradually increased with the continuous loss of agricultural labor. In order to improve the economic benefits of fruit farmers and the automation degree of orchards, it is imperative to study the intelligent picking technology. Accurate and fast fruit detection is one of the most critical steps for orchard robot automatic picking. The robustness of fruit detection in complex backgrounds and shaded environments is a key factor affecting the work of automated picking robots. This study aimed to improve the accuracy and speed of fruit detection by improving the existing methods. The results will improve the reliability of pear detection in unstructured environments and enable it to be applied to online detection tasks in an industrial computer.

Based on YOLOv5, we proposed a deep learning model YOLO-P for detecting pears in complex orchard environments. The research carried out the following design and improvements. A new module named inverted shuffle block was designed. The inverted shuffle block was used in deeper networks. Combined with the shuffle block used in the shallow networks, the backbone of YOLOv5 was reconstructed. The new backbone had a good ability to detect small targets. The activation function was replaced with Hard-Swish to reduce the computational load of the network. CBAM was inserted to improve the capture of key information. Finally, a weighted loss function was designed to further improve the feature extraction ability of small targets.

We used the Akidzuki pears as detection object of the model. We compared YOLO-P with some mainstream lightweight models. The detection effect of YOLO-P was significantly better than others. Compared with the original YOLOv5s, AP increased from 1.8% to 97.6%, and the volume of the model was compressed by 39.4% to only 8.3MB. Ablation experiments on YOLO-P demonstrated the effectiveness of these improvements. In daytime and nighttime Akidzuki pear detection experiments, we used an embedded industrial computer to test the performance of the model under different background complexities and different shade degrees. The experimental results showed that YOLO-P achieved the highest F1 score and FPS of 96.1% and 32, respectively which were 3.3% and 68.4% higher than YOLOv5s, respectively. The YOLO-P developed in this paper can provide technical support for intelligent picking in pear orchards, and can also provide a reference for other types of fruit detection in complex environments.

In this research, we only considered the situation that the degree of shade is less than 60%. In the real orchard environment, there may be fruits that are more seriously shaded and difficult to be detected. Efficiently obtain high-quality and more abundant data to train models will be our next research goal. In detection at night, border of the fruit may be similar to the environment due to the lack of light. This is one of the reasons why the accuracy at night is lower than that during the day. In follow-up research, we will consider using image enhancement algorithms to further improve the reliability of the model.

## Data availability statement

The raw data supporting the conclusions of this article will be made available by the authors, without undue reservation.

## Author contributions

HS designed the model, obtained the pear images, designed the experiments, and carried it out. BW guided the research of this paper, processed the required images, and optimized the experiment scheme. JX also guided the research, determined basic framework of the research, revised the manuscript several times, and provided the final version. All authors contributed to the article and approved the submitted version.
